# NUPR1 inhibitor ZZW-115 induces ferroptosis in a mitochondria-dependent manner

**DOI:** 10.1038/s41420-021-00662-2

**Published:** 2021-10-01

**Authors:** Can Huang, Patricia Santofimia-Castaño, Xi Liu, Yi Xia, Ling Peng, Célia Gotorbe, Jose Luis Neira, Daolin Tang, Jacques Pouyssegur, Juan Iovanna

**Affiliations:** 1 Centre de Recherche en Cancérologie de Marseille (CRCM), INSERM U1068, CNRS UMR 7258, Aix-Marseille Université and Institut Paoli-Calmettes; Parc Scientifique et Technologique de Luminy, 163 Avenue de Luminy, 13288 Marseille, France; 2 Chongqing Key Laboratory of Natural Product Synthesis and Drug Research, School of Pharmaceutical Sciences, Chongqing University, No.55 Daxuecheng South Road, 401331 Chongqing, P. R. China; 3 Aix-Marseille Université, CNRS, Centre Interdisciplinaire de Nanoscience de Marseille, UMR 7325, « Equipe Labellisée Ligue Contre le Cancer », Parc Scientifique et Technologique de Luminy, 163 Avenue de Luminy, 13288 Marseille, France; 4 Department of Medical Biology, Centre Scientifique de Monaco (CSM), 98000 Monaco, Monaco; 5 Instituto de Biología Molecular y Celular, Universidad Miguel Hernández, Edificio Torregaitán, Avda. del Ferrocarril s/n, 03202 Elche, Alicante, Spain; 6 Department of Surgery, UT Southwestern Medical Center, Dallas, TX 75390 USA; 7 CNRS, INSERM, Centre A. Lacassagne, Institute for Research on Cancer & Aging (IRCAN), University Côte d’Azur, 06107 Nice, France

**Keywords:** Pancreatic cancer, Cancer metabolism

## Abstract

Ferroptosis is an iron-dependent cell death characterized by the accumulation of hydroperoxided phospholipids. Here, we report that the NUPR1 inhibitor ZZW-115 induces ROS accumulation followed by a ferroptotic cell death, which could be prevented by ferrostatin-1 (Fer-1) and ROS-scavenging agents. The ferroptotic activity can be improved by inhibiting antioxidant factors in pancreatic ductal adenocarcinoma (PDAC)- and hepatocellular carcinoma (HCC)-derived cells. In addition, ZZW-115-treatment increases the accumulation of hydroperoxided lipids in these cells. We also found that a loss of activity and strong deregulation of key enzymes involved in the GSH- and GPX-dependent antioxidant systems upon ZZW-115 treatment. These results have been validated in xenografts induced with PDAC- and HCC-derived cells in nude mice during the treatment with ZZW-115. More importantly, we demonstrate that ZZW-115-induced mitochondrial morphological changes, compatible with the ferroptotic process, as well as mitochondrial network disorganization and strong mitochondrial metabolic dysfunction, which are rescued by both Fer-1 and N-acetylcysteine (NAC). Of note, the expression of TFAM, a key regulator of mitochondrial biogenesis, is downregulated by ZZW-115. Forced expression of TFAM is able to rescue morphological and functional mitochondrial alterations, ROS production, and cell death induced by ZZW-115 or genetic inhibition of NUPR1. Altogether, these results demonstrate that the mitochondrial cell death mediated by NUPR1 inhibitor ZZW-115 is fully rescued by Fer-1 but also via TFAM complementation. In conclusion, TFAM could be considered as an antagonist of the ferroptotic cell death.

## Introduction

The concept of ferroptosis, an iron-dependent mode of cell death, characterized by the accumulation of lipid reactive oxygen species (ROS) was first proposed in 2012 [1]. Morphologically, ferroptosis occurs mainly in cells with reduced mitochondrial size, increased bilayer membrane density, and reduction or disappearance of mitochondrial cristae [1–3]. Biochemically, ferroptotic cells usually present strong depletion of intracellular glutathione (GSH) with a concomitant decreased activity of glutathione peroxidase 4 (GPX4), leading to accumulation of Fe^2+^-dependent lipid hydroxyperoxidation, resulting in a large amount of ROS, promoting ferroptosis-cell death [2–4]. Ferroptosis may be induced by some mechanisms, such as (i) the inhibition of the cysteine import via the glutamate/cysteine antiporter (Xc-system), the reduction of GSH pool and the concomitant decrease in cell antioxidant capacity, accumulating the hydroperoxided phospholipids, and ultimately causes the occurrence of oxidative damage and ferroptotic cell death via membrane disintegration [5]; (ii) suppression of the GPX4 activity which prevents GSH-dependent of lipid hydroperoxides (L-OOH) into corresponding alcohols (L-OH). Therefore, genetic or pharmacological inhibition of GPX4 leads to the accumulation of lipid hydroperoxides, which induces ferroptosis [6, 7]. On the contrary, there are at least two key pathways antagonizing ferroptosis. The first one is controlled by the ferroptosis suppressor protein 1 (FSP1), a reductase of Co-enzyme Q10. This gene was found to be strongly related to ferroptosis by using an expression cloning or metabolic method developed to identify regulators that complement the loss of GPX4 [8, 9]. The second one was recently described by Liu et al. [10]. They identified that the stress-inducible protein NUPR1 is strongly activated in response to ferroptosis induction which in turn, induces the expression of lipocalin 2 (LCN2) which blocks the ferroptotic cell death by diminishing iron accumulation and subsequent Fenton-dependent oxidative damage.

NUPR1 is a gene described for the first time by our laboratory because it is activated during the acute phase of pancreatitis [11]. Then, it has been shown that NUPR1 is expressed in most, if not all, cancerous tissues. At the cellular level, NUPR1 has been described as participating in many processes associated with cancer, including cell cycle regulation and apoptosis, senescence, cell migration and invasion, development of metastases [12]. Importantly, NUPR1 has recently received significant attention due to its role in promoting the development and progression of PDAC [13, 14]. Some NUPR1-dependent effects are also involved in the resistance to some anticancer drugs [15–17]. The crucial role of NUPR1 as a potential therapeutic target has been previously reported since its genetic inactivation completely prevented the growth of PDAC [18]. Remarkably, other laboratories have also shown that genetic inactivation of NUPR1 stops the growth of HCC [19], non-small cell lung cancer [20], cholangiocarcinoma [21], glioblastoma [22], multiple myeloma [23], osteosarcoma [24], and more recently ovarian [25] and gastric cancer [26]. These results prompted us to identify a small inhibitor of NUPR1 to be used for treating cancers. Unfortunately, NUPR1 is an 82 residue intrinsically disordered nuclear protein (IDP) [27] and consequently, a high throughput screening, based on the principles that apply to well-folded proteins for selection of inhibitors, is inappropriate for NUPR1. Therefore, we have developed a small molecule screening by a multidisciplinary approach combining biophysics, chemistry, bioinformatics, and biology, and we have demonstrated that ZZW-115, a trifluoperazine-related compound is more effective than trifluoperazine in vitro and in vivo and without side effects [28, 29]. Treatment of PDAC xenografts, but also glioblastoma [30] and HCC [31], with ZZW-115 induces growth arrest followed by complete tumor regression. Mechanistically, ZZW-115 binds with a strong affinity to Thr68, which is located into the nuclear localization signal (NLS) of NUPR1, hampering the interaction with importins and displacing them, and therefore preventing NUPR1 to translocate from the cytoplasm to the nucleus [30]. Treatments of cancer cells with siRNA directed against NUPR1 or with ZZW-115 induce a collapse of ATP, associated with a strong reduction in OXPHOS metabolism and overproduction of ROS. The cells respond by activating glycolysis, to compensate for this energetic deficit, which rapidly consumes all energy resources, triggering necroptosis and apoptosis simultaneously [32, 29]. Altogether, these data indicate that NUPR1 inactivation with ZZW-115 is a promising anticancer strategy for PDAC, but also other cancers, and therefore to characterize its mechanism of action is clinically relevant.

In this study, we demonstrate that ZZW-115 induces a strong mitochondrial dysfunction with a ROS overproduction in combination with the collapse of the antioxidant defense system, leading to combined cell death via apoptosis and ferroptosis. Importantly, this effect is in part rescued by a forced expression of the mitochondrial factor TFAM.

## Results

### ZZW-115-induced cell death is rescued by Fer-1 and antioxidants

**Fig. 1:**
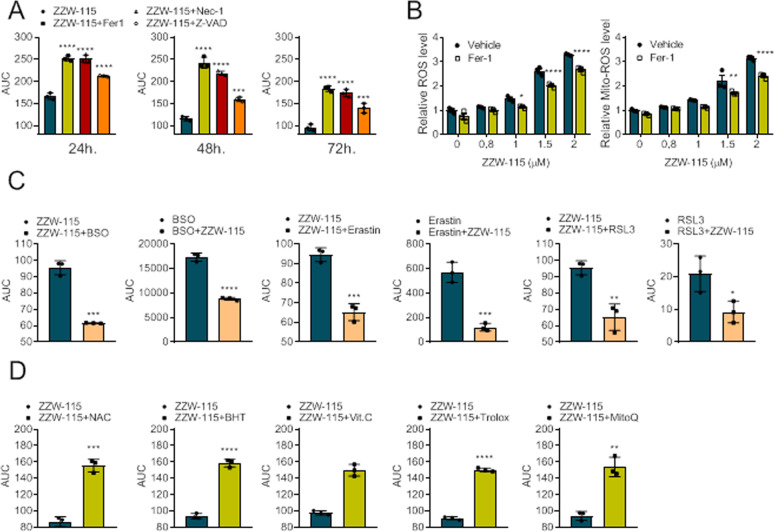
ZZW-115 induces ROS-dependent cell death in MiaPaCa-2 cells. **A** Viability upon a 24, 48, or 72 h period of treatment with increasing concentrations of ZZW-115 in MiaPaCa-2 cells in the presence or absence of Fer-1 (1 µM), Z-VAD-FMK (20 µM), or Nec-1 (40 µM). **B** ROS production in MiaPaCa-2 cells was detected using CellROX and MitoSOX Red by flow cytometry analysis after cells were incubated with indicated concentrations of ZZW-115 in the presence or absence of 1 µM Fer-1 for 72 h. **C** Chemograms were performed at increasing concentrations of ZZW-115 in combination with 5 µM BSO, or increasing concentration of BSO in combination with 0.5 µM ZZW-115; at increasing concentration of ZZW-115 in combination with 1.2 µM Erastin or increasing concentration of Erastin in combination with 0.8 µM ZZW-115; at increasing concentration of ZZW-115 in combination with 0.04 µM RSL3 or increasing concentration of RSL3 in combination with 0.8 µM ZZW-115 after 72 h of treatment. **D** Viability upon a 72 h period of treatment with increasing concentrations of ZZW-115 in MiaPaCa-2 cells in the presence or absence of NAC (20 mM), BHT (100 µM), Vitamin C (100 µM), Trolox (100 µM), or MitoQ (1 µM). AUC was calculated by integration. For each treatment, statistical significance is *P < 0.05, **P < 0.01, ***P < 0.001, ****P ≤ 0.0001 (two-way ANOVA with Sidak correction). Data represent mean ± SEM, *n* = 3 (with technical triplicates).

With the aim to determine if ZZW-115-treatment induces ferroptosis in cancer cells, we tested the potential rescue effect of Fer-1, a specific ferroptosis inhibitor [33], in ZZW-115-treated cells. MiaPaCa-2 and HepG2 as tumor cells, derived from PDAC and HCC, respectively, were challenged with increasing concentrations of ZZW-115 in the presence or not of Fer-1 (1 µM), Z-VAD-FMK (20 µM), or Nec-1 (40 µM) at 24, 48, and 72 h. As shown in Fig. 1A and Supplemental Fig. 1A, Fer-1 treatment increased cell viability upon ZZW-115 treatment. In addition, Fer-1 reduced intracellular ROS as well as specific mitochondrial ROS production in ZZW-115-treated cells (Fig. 1B and Supplemental Fig. 1B). Then, we hypothesized that the combination of ZZW-115 treatment with the drugs inducing ferroptosis or targeting antioxidant systems should increase its anticancer effect. In combination with ZZW-115, we used L-Buthionine-(S,R)-Sulfoximine BSO, a specific GCLC inhibitor; Erastin, a small molecule capable of initiating ferroptotic cell death by activating the Voltage-Dependent Anion Channels (VDAC); and RSL3, a ferroptosis activator in a VDAC-independent manner as a proof-of-concept, to treat PDAC- and HCC-derived cells. The results showed that the three drugs were able to improve ZZW-115 efficiency in both MiaPaCa-2 and HepG2 cells (Fig. 1C and Supplemental Fig. 1C). Finally, in order to determine if ROS accumulation induced by ZZW-115 treatment is involved in cell death, we performed experiments combining ZZW-115 with several unrelated antioxidants agents and measured their survival effect. Cells were treated with increasing concentrations of ZZW-115 in combination with subcytotoxic concentrations of butylated hydroxytoluene (BHT), a synthetic lipophilic organic compound, at 100 µM; NAC, a cysteine glutathione precursor, at 20 mM for MiaPaCa-2 and 15 mM for HepG2 cells; Ascorbic acid, a natural reducing agent, at 100 µM for MiaPaCa-2 and 40 µM for HepG2 cells; Trolox, a vitamin E analog, at 100 µM or MitoQ, a mitochondria-targeted antioxidant, at 1 µM for MiaPaCa-2 and 0.1 µM for HepG2 cells. Viability was systematically rescued when MiaPaCa-2 or HepG2 cells were co-treated with each of these antioxidants (Fig. 1D and Supplemental Fig. 1D). Altogether, the results confirmed that ZZW-115-induced ferroptosis is ROS-dependent, which may be prevented by ROS-scavenging agents and enhanced by inhibiting antioxidant factors.

### ZZW-115 reduces the antioxidant homeostasis in vitro and in vivo

**Fig. 2:**
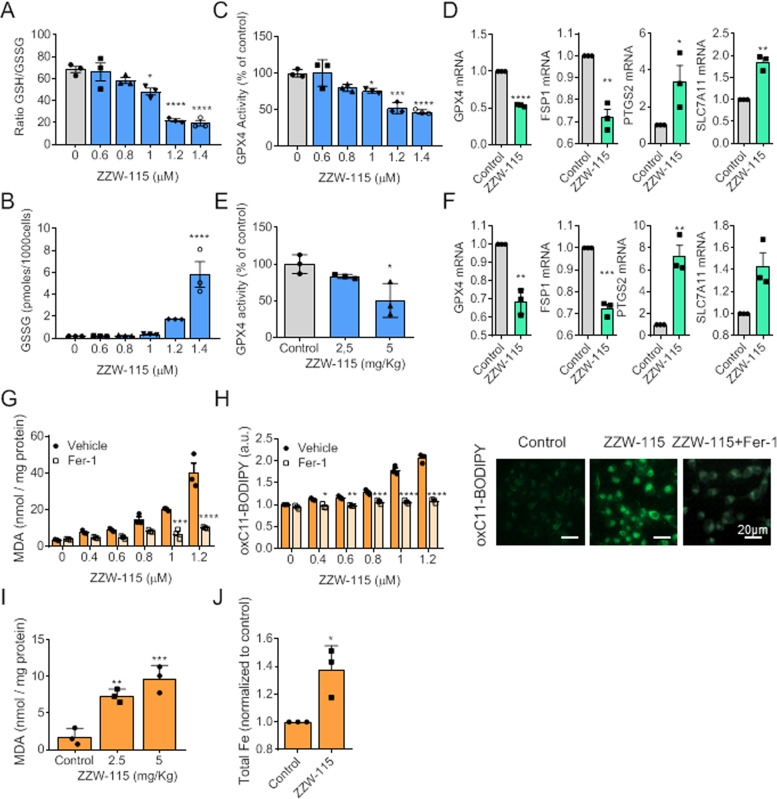
ZZW-115 impairs the antioxidant defense system and induces lipid peroxidation in MiaPaCa-2 cells, in vivo and in vitro. **A** The ratio of reduced glutathione to oxidized glutathione (GSH/GSSG), **B** GSSG content, and **C** GPX4 activity were measured in MiaPaCa-2 cells with the indicated concentration of ZZW-115 treatment for 72 h. **D** GPX4, FSP1, PTGS2, and SLC7A11 mRNA levels were measured in MiaPaCa-2 cells upon ZZW-115 treatment for 24 h and expressed as fold changes. **E** GPX4 activity was measured in MiaPaCa-2-xenografted tumors after 30 of daily treatment with different doses of ZZW-115. **F** GPX4, FSP1, PTGS2, and SLC7A11 mRNA levels were measured in MiaPaCa-2-xenografted tumors and expressed as fold changes. **G** Lipid peroxidation as malondialdehyde (MDA) levels or by the oxidation of the **H** BODIPY-C11 probe (by flow cytometry or fluorescence microscopy) were measured in cells incubated with the indicated concentration of ZZW-115, in the presence or absence of 1 µM Fer-1 for 72 h. MDA content (**I**) was measured in MiaPaCa-2-xenografted tumors after 30 of daily treatment with ZZW-115. **J** Total iron was measured in MiaPaCa-2 upon ZZW-115 treatment. For each treatment, statistical significance is *P < 0.05, **P < 0.01, ***P < 0.001, ****P ≤ 0.0001 (one-way ANOVA, Tukey’s post-hoc test, Student’s two-tailed unpaired *t*-test or two-way ANOVA with Sidak correction). Data represent mean ± SEM, *n* = 3 (with technical triplicates).

GSH plays an important role as an antioxidant molecule in cells; however, imbalances in GSH/glutathione disulfide (GSSG) ratio leads to an increased susceptibility to ROS accumulation, oxidative stress, and finally ferroptosis [10]. Taking this into account, we measured the GSSG level and calculated GSH/GSSG ratio to study whether ZZW-115-treatment alters the GSH homeostasis. Data presented in Fig. 2A, B, and Supplemental Fig. 2A, B, showed that ZZW-115 induced, in a dose-dependent manner, a decreasing of the reduced/oxidized glutathione ratio with a strong increase of the intracellular GSSG level, in both cellular models. Moreover, we have also studied the activity of GPX4, an antioxidant enzyme that neutralizes lipid peroxides and protects membrane fluidity. As shown in Fig. 2C and Supplemental Fig. 2C, a decrease of GPX4 activity was observed in both cell types in a dose-dependent manner. Then, we studied the mRNA levels by qRT-PCR analysis of GPX4 and key genes involved in ferroptosis, such as FSP1, which confers protection against ferroptosis elicited by GPX4 deletion [8]; PTGS2, the prostaglandin-endoperoxide synthase 2, a key enzyme in prostaglandin biosynthesis [34]; or SLC7A11, a member of the cystine/glutamate transporter system [1]. We found that ZZW-115-treatment dramatically dysregulated the expression of these genes in both cells (Fig. 2D and Supplemental Fig. 2D). The induction of ferroptosis could be considered a promising therapeutic approach for treating resistant tumors [35]. We have recently demonstrated a strong anticancer effect of ZZW-115 in a panel of xenografted human tumors in vivo [29, 30]. However, whether the ferroptosis induced by ZZW-115 participates in this effect is currently unknown. We induced xenografts with MiaPaCa-2 and HepG2 cells in nude mice and treated them for 4 or 3 weeks, respectively, with vehicle alone and 2.5 or 5.0 mg/kg/day of ZZW-115. Then, we measured the GPX4 activity (Fig. 2E and Supplemental Fig. 2E) and analyzed the mRNA levels of the key genes involved in ferroptosis by qRT-PCR analysis (Fig. 2F and Supplemental Fig. 2F). Consistent with the in vitro results, we found that GPX4 activity was significantly decreased and the mRNA expression was dysregulated upon ZZW-115-treatment. Together, these results suggest that the key antioxidant systems fail to protect cells against oxidative damage induced by ZZW-115.

### Lipid peroxidation is increased upon ZZW-115 in vitro and in vivo

To further explore the molecular mechanisms of ZZW-115 in inducing ferroptosis, we analyzed lipid peroxidation, which is an important signaling event in activating ferroptosis. Lipid peroxidation is indispensable for ferroptosis, and GPX4 prevents ferroptosis through clearance of the lipid peroxides [34]. Malondialdehyde (MDA) is one of the most important end-products of lipid peroxidation; therefore, we tested whether ZZW-115-treatment increases MDA accumulation in PDAC and HCC cells. As shown in Fig. 2G and Supplemental Fig. 2G, ZZW-115 induced a dose-dependent accumulation of MDA in both cell lines and this effect was significantly suppressed by Fer-1. In addition, we performed flow cytometry analysis and fluorescence microscopy after staining the cells with BODIPY 581/591 C11. The result showed that ZZW-115-treatment induced the accumulation of lipid hydroperoxide in cells, an effect that can be prevented by Fer-1 co-treatment (Fig. 2H and Supplemental Fig. 2H). Moreover, lipid peroxidation product MDA was increased in both tumor tissues under ZZW-115 treatment in a dose-dependent manner as shown in Fig. 2I and Supplemental Fig. 2I. Thus, we conclude that ZZW-115 also induces ferroptosis in vivo. In addition, previous studies demonstrated that the accumulation of iron is a key mediator of cytotoxicity in ferroptosis. We explored the level of intracellular concentration of iron in MiaPaCa-2 cells treated with ZZW-115 for 24 h and found a significant increase in iron accumulation (Fig. 2J).

### ZZW-115 induces mitochondrial dysfunction by ROS overproduction

**Fig. 3:**
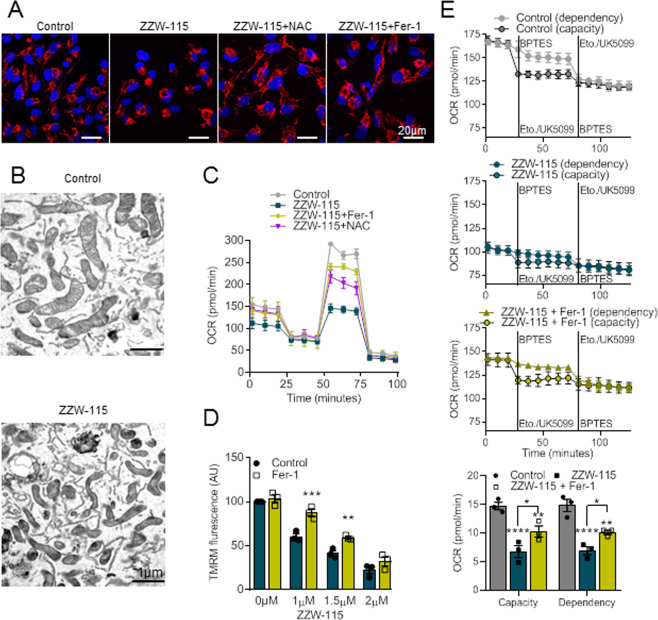
ROS scavengers rescue ZZW-115-induced mitochondrial dysfunction in ferroptosis. **A** The mitochondrial network in MiaPaCa-2 cells with 1.4 µM ZZW-115 treatment in the presence or absence of 1 µM Fer-1 or 10 mM NAC for 72 h. **B** Representative transmission electron microscopy images of MiaPaCa-2 cells with 1.4 µM ZZW-115 treatment for 24 h are shown. **C** Mitochondrial oxygen consumption rate (OCR) was determined in MiaPaCa-2 cells upon 1.4 µM ZZW-115 treatment in the presence or absence of 1 µM Fer-1 or 10 mM NAC for 72 h. **D** TMRM fluorescence was measured by flow cytometry analysis of MiaPaCa-2 cells treated with indicated concentration of ZZW-115 alone or in the presence of 1 μM Fer-1. **E** Glutamine dependency and capacity in MiaPaCa-2 cells were measured after 72 h of treatment with 1.4 µM ZZW-115 alone or in the presence of 1 μM Fer-1. For each treatment statistical significance is *P < 0.05, **P < 0.01, ***P < 0.001, ****P ≤ 0.0001 (two-way ANOVA with Sidak correction or one-way ANOVA, Tukey’s post-hoc test). Data represent mean ± SEM, *n* = 3 (with technical triplicates).

In our previous studies, we demonstrated that NUPR1 inactivation was associated with a strong mitochondrial dysfunction [31, 32, 36]. Here we investigated the effect of antioxidants and Fer-1 treatment on mitochondria of MiaPaCa-2 cells treated with ZZW-115. Using the MitoTracker red to determine the cellular mitochondrial network, we observed that ZZW-115 treatment induces strong disorganization, which agrees with the pictures obtained with transmission electron microscopy (TEM). Of note, this mitochondrial network disorganization was completely rescued by the treatment with NAC or Fer-1 as presented in Fig. 3A. As shown in Fig. 3B, treatment with ZZW-115 induced strong morphological changes of these organelles with an obvious decrease in their volume compared to normal mitochondria, increased membrane density and an important reduction or disappearance of mitochondrial cristae as explored by TEM. Importantly, all these morphological features are usually observed in ferroptotic cells [34]. We then studied the OXPHOS activity of the mitochondria after treatment with ZZW-115 alone or in combination with NAC or Fer-1. As expected, ZZW-115-treatment induced a strong decreased oxygen consumption rate (OCR), particularly in the maximal respiratory capacity, which was rescued by NAC or Fer-1 as showed in Fig. 3C. High mitochondrial membrane potential (MMP) is required for mitochondrial ATP production and OXPHOS, which could be disrupted by lipid peroxidation or high ROS level, thereby resulting in cascade amplification in cells [37]. To test this possibility, we monitored MMP by using TMRM staining upon ZZW-115 and Fer-1 treatment. As expected, ZZW-115 decreased MMP in a dose-dependent manner, which was inhibited by Fer-1 as shown in Fig. 3D. Furthermore, because glutamine metabolic reprogramming is required for fuel supply of glutathione and redox homeostasis in cancer cells during ferroptosis [38, 39], we studied the glutamine oxidation pathway in mitochondria after treatment with ZZW-115 alone or in combination with Fer-1. We found a dramatic decrease in glutamine capacity and dependency upon ZZW-115 treatment, the effect that was reversed by Fer-1 as showed in Fig. 3E. Altogether, these results demonstrate that ZZW-115 induces morphological changes in mitochondria, compatible with ferroptotic features, as well as strong mitochondria dysfunction, that can be rescued by both Fer-1 and antioxidant agents. It suggests that these morphological and functional mitochondrial changes are, at least in part, downstream of the ROS production.

### ZZW-115 induces changes in mitochondrial master genes

Among the mitochondria-related genes previously described as deregulated after inactivation of NUPR1, such as LONP1, PINK1, NRF1, and TFAM [32], TFAM downregulation seems to be a promising candidate responsible for mitochondrial dysfunction since it is a key regulator of mitochondrial biogenesis. In fact, TFAM is a core mitochondrial transcription factor, responsible for recruiting mitochondrial RNA polymerase and transcription factor T2BM to activate transcription [40]. Additionally, TFAM is an abundant protein that coats and packages mitochondrial DNA forming the mitochondrial nucleoid [41]. Remarkably, TFAM acts as an antioxidant factor under strong oxidative stress conditions in fly [42] and mammalian cells through the inactivation of the pro-inflammatory factor NFAT [43].

**Fig. 4:**
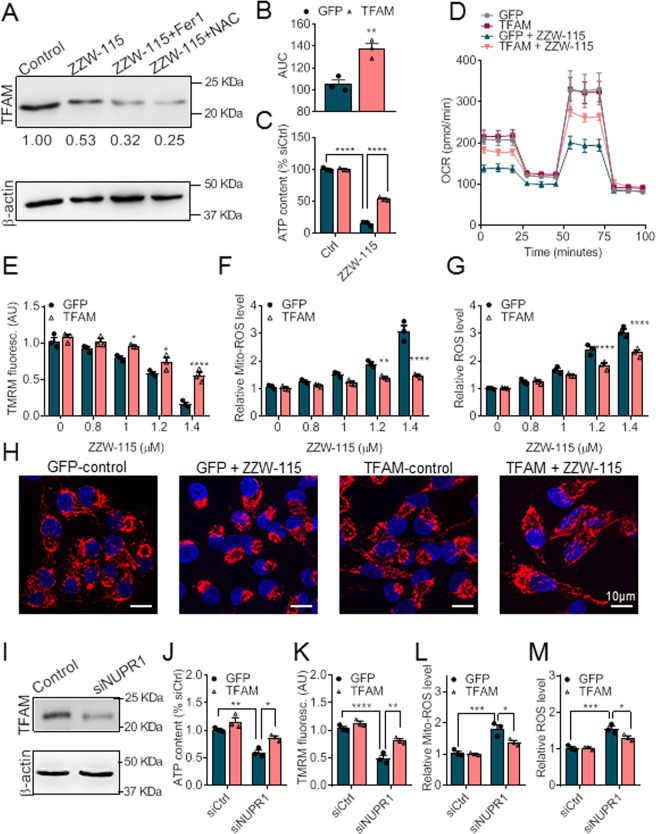
TFAM acts as a mediator of ZZW-115-induced ferroptosis. **A** Western blot analysis of TFAM expression in MiaPaCa-2 cells with 1.4 µM ZZW-115 in the presence or absence of 1 μM Fer-1 or NAC for 72 h. **B** Cell viability in TFAM- or control-transfected MiaPaCa-2 cells following treatment with ZZW-115 for 72 h. AUC was calculated by integration. **C** ATP content in TFAM- or control-transfected MiaPaCa-2 cells following treatment with ZZW-115 for 72 h. **D** OCR was determined in TFAM- or control-transfected MiaPaCa-2 cells upon 1.4 µM ZZW-115 treatment for 72 h. TMRM fluorescence (**E**), Mito-ROS level (**F**), and cellular ROS level (**G**) were measured in Indicated MiaPaCa-2 cells following treatment with indicated concentration of ZZW-115 for 72 h. **H** The mitochondrial network in indicated MiaPaCa-2 cells with 1.4 µM ZZW-115 treatment for 72 h. **I** Western blot analysis of TFAM expression in siNUPR1-RNA-transfected cells. ATP content (**J**) TMRM fluorescence (**K**), Mito-ROS level (**L**), and cellular ROS level (**M**) were measured in siNUPR1-transfected cells. For each treatment, statistical significance is *P < 0.05, **P < 0.01, ***P < 0.001, ****P ≤ 0.0001 (two-way ANOVA with Sidak correction or Student’s two-tailed unpaired *t*-test). Data represent mean ± SEM, *n* = 3 (with technical triplicates).

We hypothesized that the downregulation of TFAM could be a mediator of the ferroptotic cell death induced by ZZW-115. First, we measured the TFAM protein levels in MiaPaCa-2 cells treated with ZZW-115 alone or together with Fer-1 or NAC. As presented in Fig. 4A, treatment with ZZW-115 decreased to 53 ± 13% of the TFAM protein compared to control cells. Importantly, treatment with Fer-1 or NAC did not prevent this decrease indicating that the ZZW-115 effect on TFAM level is mediated by NUPR1 inhibition, rather than ROS induced by ZZW-115 treatment. Then, we overexpressed TFAM through a plasmid transfection followed by challenging with a dose-response treatment of ZZW-115 and found a significant rescue in terms of cell survival, ATP production and OXPHOS capacity, as shown in Figs. 4B, C, and D. We also measured the MMP, the mitochondrial ROS and total ROS production, and in response to increasing dose of ZZW-115 on TFAM-transfected cells and found that all these biological parameters were strongly improved by TFAM as presented in Figs. 4E, F, and G. Finally, we analyzed the mitochondrial network in both, GFP- and TFAM-transfected, cells in response to the ZZW-115 treatment and found that the mitochondrial network disorganization induced by ZZW-115 was completely rescued by TFAM complementation (Fig. 4H). Moreover, in order to demonstrate that the previous results are the consequence of the inhibition of NUPR1 by ZZW-115, we used NUPR1-depleted MiaPaCa-2 cells by a specific NUPR1-siRNA. Upon NURP1 inhibition, TFAM expression was downregulated, as shown in Fig. 4I. Interestingly, TFAM overexpression, in NUPR1-depleted cells, was able to rescue the ATP content (Fig. 4J), the MMP (Fig. 4K), as well as to decrease the mitochondrial and cellular ROS level (Fig. 4L and M, respectively). Altogether, these results demonstrate that mitochondrial cell death induced by ZZW-115 treatment or by inhibiting siNUPR1 treatment is rescued by TFAM complementation. Consequently, TFAM could be considered as an antagonist of the ferroptotic cell death regulated by NUPR1.

## Discussion

In this work, we describe a parallel induction of apoptosis (Z-VAD-FMK sensitive) and a genuine ferroptotic cell death pathway (Fer-1 sensitive) launched by the NUPR1 inhibitor ZZW-115. This oxidative pathway induces downregulation of the biogenic mitochondrial factor TFAM, a strong mitochondrial dysfunction with high ROS production and lipid hydroperoxidation, and a concomitant fail of the key endogenous antioxidant systems, which can be reversed by TFAM complementation. Consequently, TFAM is in part an antagonist of ferroptotic-induced cell death.

It has been recently established a central role of NUPR1 stress protein against ferroptosis acting as a transcription inductor of LCN2 [10]. Notably, NUPR1 is also involved in resistance to other cell deaths such as apoptosis and necroptosis [29] and its expression is associated with the resistance to several drugs [16]. In this work, we demonstrated that inhibition of the NUPR1 by ZZW-115 induces ferroptosis which is reversed by Fer-1 and ROS scavengers and, most important, by TFAM complementation. It is important to note that NUPR1 inactivation induced downregulation of TFAM expression. Because NUPR1 acts as a transcriptional regulator, we investigated whether downregulation of TFAM was directly regulated by NUPR1 inactivation or indirectly thought the consequent ROS production. Remarkably, this effect was undoubtedly induced by a direct effect since antioxidants treatment did not prevent this downregulation as demonstrated in Fig. 4A. It is important to note that ROS-induced production by ZZW-115 treatment is responsible for the strong mitochondrial network disorganization and mitochondrial dysfunction since it is reversed by Fer-1 and ROS scavengers. TFAM downregulation seems to play a key role in this process since, on one hand, it is directly downregulated by NUPR1 inactivation and, on the other hand, its complementation reverses the mitochondrial dysfunctions, network organization, and ROS production. All in all, our data indicate that mitochondrial cell death mediated by TFAM downregulation is central in cell death by ferroptosis.

Another important point to be noted is that, concomitantly to the increased ROS accumulation, lipid hydroperoxidation, and elevated iron levels found in cells treated with ZZW-115, we observed a dramatic fail in the key endogenous antioxidant systems such as GSH/GSSG ratio and GPX4 activity and expression of key genes involved in ferroptosis. Altogether, we showed that NUPR1 inactivation induces the accumulation of ROS with a concomitant decrease of antioxidant mechanisms.

Remarkably, in vivo treatment of PDAC- and HCC-derived xenograft showed a dose-dependent effect on GPX4 activity and lipid peroxidation indicating that ZZW-115 induced tumor growth arrest by, at least in part, ferroptosis. Inducing ferroptosis is considered a promising strategy to treat aggressive cancers. For example, several ferroptotic agents are in evaluation for PDAC such as artesunate (ART) [44], the combination of cotylenin A (CN-A) and phenylethyl isothiocyanate (PEITC) [45], or the combination of piperlongumine (PL), CN-A and sulfasalazine [46]. Also, in HCC this strategy is in evaluation. Sorafenib, a tyrosine kinase inhibitor widely used in the treatment of advanced HCC, induces ferroptosis of HCC as part of its biological effects [47]. In addition, inhibition of sigma 1 receptor (S1R), which is abundantly expressed in hepatocytes, also promotes ferroptosis in HCC cells [48]. Other anticancer approaches are able to induce ferroptosis in HCC [49]. However, PDAC, as well as HCC, are resistant tumors that express a high level of NUPR1 [50], which may justify the failure of this approach. Therefore, a promising strategy to improve this treatment could be the association of ferroptosis-inducing agents with NUPR1 inhibitors like ZZW-115.

Several defense mechanisms protect against ferroptosis have been reported but that mediated by NUPR1 deserves particular attention. On one hand, its activation in response to ferroptotic agents mediates the activation of the LCN2 [10], which acts directly against ferroptosis. Furthermore, an additional and complementary system is reported in this work in which NUPR1 inactivation mediated the downregulation of the TFAM. Interestingly, NUPR1 is a stress-induced protein suggesting that its role is exclusively under stress conditions. How TFAM acts against ferroptosis is suggested by its antioxidant effect but we cannot exclude another additional effect at this time.

## Materials and methods

### Cell lines and cell culture

Cell lines (MiaPaCa-2 cells and HepG2 cells) were obtained from American Type Culture Collection (ATCC, USA) and cultured in Dulbecco’s modified Eagle’s medium (DMEM) containing 10% fetal bovine serum (Lonza, Basel, Switzerland) in an incubator with 5% CO_2_ at 37 °C. Z-VAD-FMK (Z-VAD), Necrostatin-1 (Nec-1), Ferrostatin-1 (Fer-1), Trolox, Butylated hydroxytoluene (BHT), Ascorbic Acid (Vitamin C), N-acetyL-cysteine (NAC), and L-Buthionine-(S,R)-Sulfoximine (BSO) were obtained from Merk; and Mitoquinone (MitoQ) was obtained from mesylate Selleck Chemicals.

### Cell viability

Cell viability was determined by crystal violet assay. Cells were plated in triplicate in 96-well plates allowed to attach overnight, then incubated with various concentrations of ZZW-115 in the presence or absence of inhibitors at the indicated time. Medium was discarded, cells were fixed with 1% glutaraldehyde solution, washed with PBS and stained with 0.1% crystal violet solution in 70% methanol. After discarding the crystal violet solution, cells were washed with PBS three times and 1% SDS solution was added to solubilize the stain. Absorbance was read at 590 nm on Epoch™ Microplate Spectrophotometer. AUC values were calculated by nonlinear regression curves with a robust fit using GraphPad software.

### qRT-PCR

Total RNA was extracted from cells using Trizol kit (Invitrogen, Carlsbad, CA, USA) and cDNA was obtained by reverse-transcribed using Go Script kit (Promega), according to the manufacturer’s instructions. Real-time quantitative PCR (qRT-PCR) was performed using Stratagene MXPro-MX3005P system. Primer sequences are listed below: GPX4-F: 5′-CCTGGACAAGTACCGGGGCT-3′; GPX4-R: 5′-AAACCACACTCAGCGTATCG-3′; SLC7A11-F: 5′-CAGCTGTGGGCATAACTGTA-3′; SLC7A11-R: 5′-ATTGCTGTGAGCTTGCAAAA-3′; PTGS2-F: 5′-CGCTCAGCCATACAGCAAAT-3′; PTGS2-R: 5′- CCGGGTACAATCGCACTTAT-3′; FSP1-F: 5′-ACATGGTGAGGCAGGTCCA-3′; FSP1-R: 5′-GCCACTTGGGAGTGAATGAG-3.

### Xenografts

Xenografts were induced with MiaPaCa-2 and HepG2 cells and treated with 2.5 or 5 mg/kg/day as previously described [29, 31].

### GPX4 activity assay

Glutathione peroxidase activity assay kit (Abcam, #ab102530, Cambridge, MA) was used to determine the activity of GPX4. It was based on the oxidation of glutathione (GSH) to oxidized glutathione disulfide (GSSG) catalyzed by GPX4, which was then recycled back to GSH using glutathione reductase and NADPH. The oxidation of NADPH to NADP+ indicated GPX4 activity. In brief, 5 × 10^5^ cells were reseeded in 10 cm cell culture dishes for attachment overnight and then treated with the indicated concentrations of ZZW-115 for 72 h. Cells or 100 mg tumor tissues were harvested, washed, resuspended in cold assay buffer. Cells were homogenized quickly by pipetting up and down and tumors were homogenized with a Dounce homogenizer. Supernatants were collected and kept on ice after centrifuge. Samples were mixed with reaction reagent, following manufacturer’s instruction and we measured the OD at 340 nm. Then, cumene hydroperoxide solution was then added to the samples. The enzymatic reaction was run in 96-well plates and NADPH oxidation was monitored by OD at 340 nm over 5 m at 25 °C on a FLUOstar Omega plate reader.

### Measurement of ROS and mitochondrial ROS

Cells were seeded at 8 × 10^4^ cells per well in 24-well plates. The next day, cells were treated with indicated concentrations of ZZW-115 alone or in the presence of 1 μM Fer-1 for 72 h. After that, cells were incubated with 5 μM CellROX Green Reagent (C10444, Thermo, USA) or 10 μM MitoSOX Red (M36008, Thermo, USA) at 37 °C for 30 m in the dark. Then, the unincorporated dye was removed by washings with prewarmed PBS. Samples were then harvested by accutase, centrifuged at 1500 rpm for 5 m and the pellets were resuspended in 200 µL prewarmed HBSS (Gibco, Life Technologies) for flow cytometry. 10,000 events per sample were collected in a MACSQuant-VYB, and data were analyzed with FlowJo software.

### Measurement of OXPHOS and glycolysis

Cells were plated at 24-well plates (Seahorse) and incubated overnight in Standard DMEM. Cells were treated with ZZW-115 (1 μM) alone or in the presence of Fer-1 (1 μM) or NAC (10 mM) for 72 h. The Oxygen Consumption Rate (OCR) (pmol O2/min) and Extracellular Acidification Rate (ECAR) ECAR (mPH/min) were measured using the Seahorse Bioscience XF24 Extracellular Flux Analyzer. Before the measurement of OCR or ECAR, cells were incubated in XF assay medium supplemented with 2 mM L-glutamine, with or without 10 mM glucose, with or without 1 mM pyruvate in a 37 °C non-CO_2_ incubator for 1 h. OCR measurement is under basal conditions in response to 1 μM oligomycin, 0.5 µM rotenone (Millipore Sigma), and 0.25 μM or 0.5 μM carbonylcyanide p-(trifluoro-methoxy)phenylhydrazone (FCCP) in MiaPaCa-2 or HepG2 cells, respectively. ECAR measurement was measured under basal conditions and in response to 1 μm oligomycin, 10 mM glucose and 100 mM 2-deoxyglucose (2DG). The rate of glutamine fuel oxidation was determined by the Seahorse XF mito fuel flex test. glutaminase inhibitors (BPTES 3 μM), carnitine palmitoyl-transferase 1 A (Etomoxir 4 μM), and glucose oxidation (UK5099 2 μM) were used in the test. Glutamine capacity and dependency were calculated accordingly to the manufacturer’s instructions. The OCR and ECAR values were calculated normalized with the number of cells.

### Lipid peroxidation assay

MDA lipid peroxidation assay kit (ab118970, ABCAM, Cambridge, UK) was used according to the manufacturer’s specifications. For determining MDA production in MiaPaCa-2 and HepG2 xenografts, 10 mg of tumor tissue was used. For cell experiments, 5 × 10^5^ cells were reseeded in 10-cm cell culture dishes and allowed to attach overnight. Then, cells were incubated with the indicated concentration of ZZW-115 and Fer-1 for 72 h. Tumor tissues and harvested cells were homogenized in lysis solution and centrifugated to recover the supernatant. The equivalent amount of protein was used and the thiobarbituric acid solution was added at 95 °C and incubated for 1 h. Then, samples were cooled to room temperature in an ice bath for 10 m. Fluorescence was read at Ex/Em = 532/553 nm on a TECAN infinite 96-plate reader.

### Detection of lipid hydroperoxides

cells were seeded in 12-well plates at a density of 2.5 × 10^4^ cells per well. The next day, cells were treated with indicated concentrations of ZZW-115 alone or in the presence of 1 μM of Fer-1 for 72 h. After that, cells were incubated in 200 µL fresh medium for 30 m, containing 2 µM BODIPY™ 581/591 C11 (Invitrogen Molecular Probes, D3861) at 37 °C. Then, cells were washed two times with PBS. For cytometry experiments, Samples were then harvested by accutase, centrifuged at 1500 rpm for 5 m and the pellets were resuspended in 200 µL prewarmed HBSS (Gibco, Life Technologies) for flow cytometry. 10,000 events per sample were collected in a MACSQuant-VYB, and data were analyzed with FlowJo software. For fluorescence microscopy experiment, imaging acquisition was directly performed on a Zeiss Axio Imager Z2 microscope.

### Glutathione assay

GSH/GSSG-Glo assay kit (V6611, Promega) was used following manufacturer’s protocol. In brief, 5000 cells per well were seeded overnight in 96-well plates. Cells were treated with indicated concentrations of ZZW-115 for 72 h in triplicates. Total intracellular glutathione and GSSG were measured. Luciferin Generation Reagent and Detection Reagent were added to all wells, respectively, assays were mixed and then luminescence was measured using the Tristar multimode microplate reader. GSSG and total glutathione concentration were calculated using a glutathione standard curve and normalized by the cell number. GSH/GSSG ratios were calculated using the following equation: GSH/GSSG = [Total GSH-(2 × GSSG)]/GSSG.

### Electron microscopy

Cells were prepared according to the NCMIR protocol for SBF-SEM. Seventy nanometers ultrathin sections were cut using a Leica UCT Ultramicrotome (Leica, Austria) and deposited on formvar-coated slot grids. Samples were observed in an FEI Tecnai G2 at 200 KeV and acquisition of imagine was performed on a Veleta camera (Olympus, Tokyo, Japan).

### Mitochondrial network

Mitochondrial network localization was performed by incubation of cells for 30 m at 37 °C with MitoTracker DeepRed FM (200 nM, Molecular Probes). Subsequently, cells were washed and fixed with 4% paraformaldehyde for 10 m. Finally, samples were mounted using the Prolong Gold antifade reagent with DAPI. Confocal images were acquired using an inverted microscope equipped with LSM 880 controlled by Zeiss Zen Black software.

### Mitochondrial membrane potential assay

Mitochondrial membrane potential assay was performed using MitoProbe TMRM Assay Kit (M20036, Invitrogen) following the manufacturer’s protocol. After incubation, cells were dissociated using accutase and resuspended in 200 μL PBS at the density of 1 × 10^6^ cells/mL. Add 1 μL of 20 μM stock TMRM reagent solution to the cells and incubate for 30 m at 37 °C, 5% CO_2_. Data were analyzed on flow cytometry with 561 nm excitation. Ten thousands events per sample were collected in a MACSQuant-VYB (Miltenyi Biotec, Surrey, UK). Data analysis was performed using the FlowJo software.

### Western blot

Protein extracts were resolved by SDS-PAGE and then transferred onto the nitrocellulose membranes for 1 h. Then, membranes were blocked for 1 h at room temperature with TBST (tris-buffered saline), 5% BSA, and blotted overnight in TBST 5% BSA containing primary antibodies at 1:500 overnight with corresponding antibodies at 4 °C. Subsequently, the blot was washed and incubated with HPR-conjugated secondary antibody (Boster, Pleasanton CA, USA) for 1 h at room temperature at 1:5000 before being revealed with ECL (enhanced chemo-luminescence). The acquisition was performed by a Fusion FX7 imagine system (Vilber-Lourmat, Sud Torcy, France). The following primary antibodies were used: rabbit polyclonal TFAM (# 7495, cell signaling), mouse monoclonal β-actin (#A5316, Sigma).

### Plasmid transfection

5 × 10^5^ cells were seeded in six-well plates overnight and transfected with 2.5 μg pcDNA3 TFAM-mClover [51] (Addgene plasmid # 129574) or control GFP plasmid, using Lipofectamine 3000 transfection reagent (Thermo Fisher Scientific) in each well, following the manufacturer’s protocol as described for DNA.

### siRNA transfection

Cells were plated at 70% confluence and INTERFErin™ reagent (Polyplus-transfection) was used to perform siRNA transfections, following manufacturer’s protocol. Scrambled siRNA that targets no known gene sequence was used as a negative control. The sequence of Nupr1-specific siRNA was r(GGAGGACCCAGGACAGGAU)dTdT.

### ATP content

ATP content was measured using the CellTiter-Glo Assay kit (G7571, Promega). Five thousands cells per well were seeded overnight in 96-well plates and treated for the indicated concentration of ZZW-115 alone or in the presence of 1 μM Fer-1 for 72 h in triplicates. One hundred microliters CellTiter-Glo reagents were added to each well. Mix content for 2 m and then incubate at room temperature for 10 m. The luminescence was recorded under the Tristar multimode microplate reader.

### Iron levels

The intracellular iron concentration was measured using the iron assay kit (MAK025, SIGMA-ALDRICH) following the manufacturing instructions with small modifications. Briefly, 5 × 10^6^ MiaPaCa-2 cells were homogenized in 200 µl of Iron Assay Buffer and centrifuge at 16,000 × *g* for 10 m at 4 °C to remove insoluble material. Following, 75 µl samples were added in a 96-well plate and the volume were brought to 100 µl per well with Assay Buffer. Five microliters of Iron Reducer were added to each well to reduce Fe^3+^ to Fe^2+^. The plate was incubated for 30 m at room temperature and protected from light. After incubation, 100 µl of Iron Probe were added to each well and incubated for 60 m at room temperature and protected from light. The absorbance was measured at 593 nm. Iron concentrations were evaluated from an iron standard curve and normalized by the number of cells. The data are represented as total iron concentration (µM)/ number of cells.

### Statistics

Statistical analyses were conducted by using the unpaired two-tailed Student *t*-test, or one-way ANOVA with Tukey’s post-hoc test or two-way ANOVA with Sidak correction. The results were expressed as the mean ± SEM of at least three independent experiments. A *p*-value of <0.05 was regarded as statistically significant.

## Supplementary information

Legend of Supplementary Figures

Supp Figure 1

Supp Figure 2

## Data Availability

All the data used during the study are available from the corresponding author on request.
