# Supplementary material for: NUPR1 inhibitor ZZW-115 induces ferroptosis in a mitochondria-dependent manner
Source: Cell Death Discov. 2021 Oct 1;7:269. doi: 10.1038/s41420-021-00662-2 (PMC8486797; doi:10.1038/s41420-021-00662-2)
Supplement: Supplementary file 1 — Legend of Supplementary Figures [file 41420_2021_662_MOESM1_ESM.docx]

**Legend of Supplementary Figures**

**Supplementary Figure 1**

**ZZW-115 induces ROS-dependent cell death in HepG2 cells.** (A) Viability upon a 24, 48 or 72-h period of treatment with increasing concentrations of ZZW-115 in HepG2 cells in the presence or absence of Fer-1 (1 µM), Z-VAD-FMK (20 µM) or Nec-1 (40 µM), from left to right. (B) ROS production in HepG2 cells was detected using CellROX and MitoSOX Red by flow cytometry analysis after cells were incubated with the indicated concentration of ZZW-115 in the presence or absence of 1 µM Fer-1 for 72 h. (C) Chemograms were performed at increasing concentrations of ZZW-115 in combination with 5 µM BSO, or increasing concentration of BSO in combination with 0.5 µM ZZW-115; at increasing concentration of ZZW-115 in combination with 4 µM Erastin or increasing concentration of Erastin in combination with 0.8 µM ZZW-115; at increasing concentration of ZZW-115 in combination with 0.03 µM RSL3 or increasing concentration of RSL3 in combination with 0.8 µM ZZW-115 after 72 h of treatment. (D) Viability upon a 72-h period of treatment with increasing concentrations of ZZW-115 in HepG2 cells in the presence or absence of NAC (15 mM), BHT (100 µM), Vitamin C (100 µM), Trolox (40 µM) or MitoQ (0.1 µM). AUC was calculated by integration. For each treatment, statistical significance is *P < 0.05, **P < 0.01, ***P < 0.001, ****P ≤ 0.0001 (2-way ANOVA with Sidak correction). Data represent mean ± SEM, n = 3 (with technical triplicates).

**Supplementary Figure 2**

**ZZW-115 impairs the antioxidant defense system and induces lipid peroxidation in HepG2 cells, *in vivo* and *in vitro*.** (A) The ratio of reduced glutathione to oxidized glutathione (GSH/GSSG), (B) GSSG content and (C) GPX4 activity were measured in HepG2 cells with the indicated concentration of ZZW-115 treatment for 72 h. (D) GPX4, FSP1, PTGS2 and SLC7A11 mRNA levels were measured in HepG2 cells upon ZZW-115 treatment for 24 h and expressed as fold changes. (E) GPX4 activity was measured in HepG2-xenografted tumors after 30 of daily treatment with different doses of ZZW-115. (F) GPX4, FSP1, PTGS2 and SLC7A11 mRNA levels were measured in HepG2-xenografted tumors and expressed as fold changes. (G) Lipid peroxidation as malondialdehyde (MDA) levels or by the oxidation of the (H) BODIPY-C11 probe (by flow cytometry or fluorescence microscopy) were measured in cells incubated with the indicated concentration of ZZW-115, in the presence or absence of 1 µM Fer-1 for 72 h. MDA content (I) was measured in HepG2-xenografted tumors after 30 of daily treatment with ZZW-115. For each treatment, statistical significance is *P < 0.05, **P < 0.01, ***P < 0.001, ****P ≤ 0.0001 (1-way ANOVA, Tukey’s post hoc test, Student’s 2-tailed unpaired t test or 2-way ANOVA with Sidak correction). Data represent mean ± SEM, n = 3 (with technical triplicates).
