# Supplementary figures and images for: NUPR1 inhibitor ZZW-115 induces ferroptosis in a mitochondria-dependent manner
Source: Cell Death Discov. 2021 Oct 1;7:269. doi: 10.1038/s41420-021-00662-2 (PMC8486797; doi:10.1038/s41420-021-00662-2)

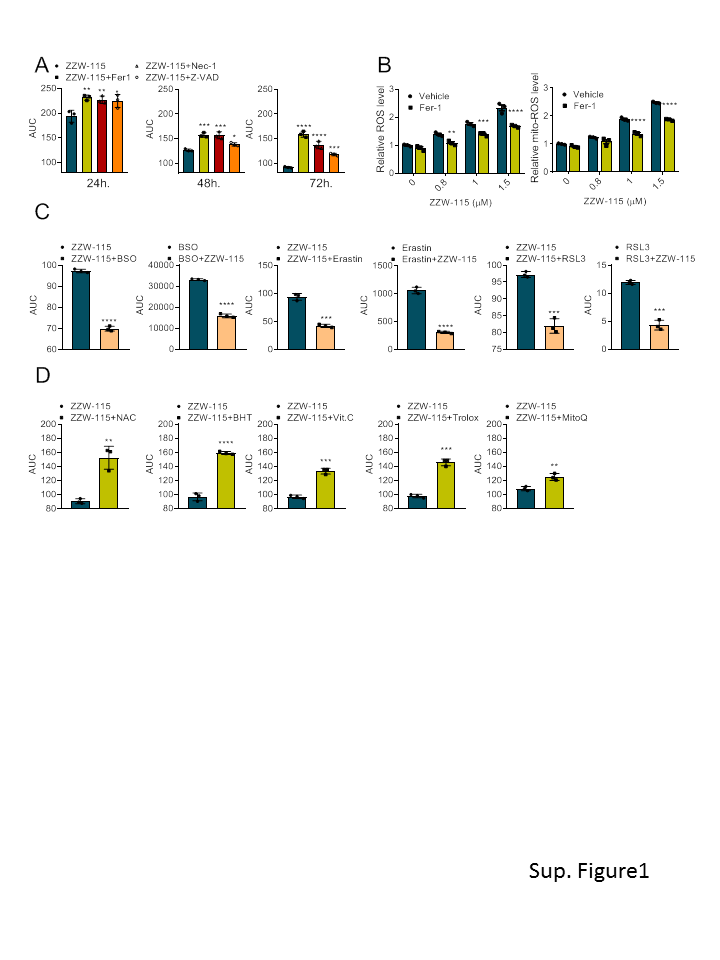

Supplement: Supplementary file 2 — Supp Figure 1 [file 41420_2021_662_MOESM2_ESM.tif]

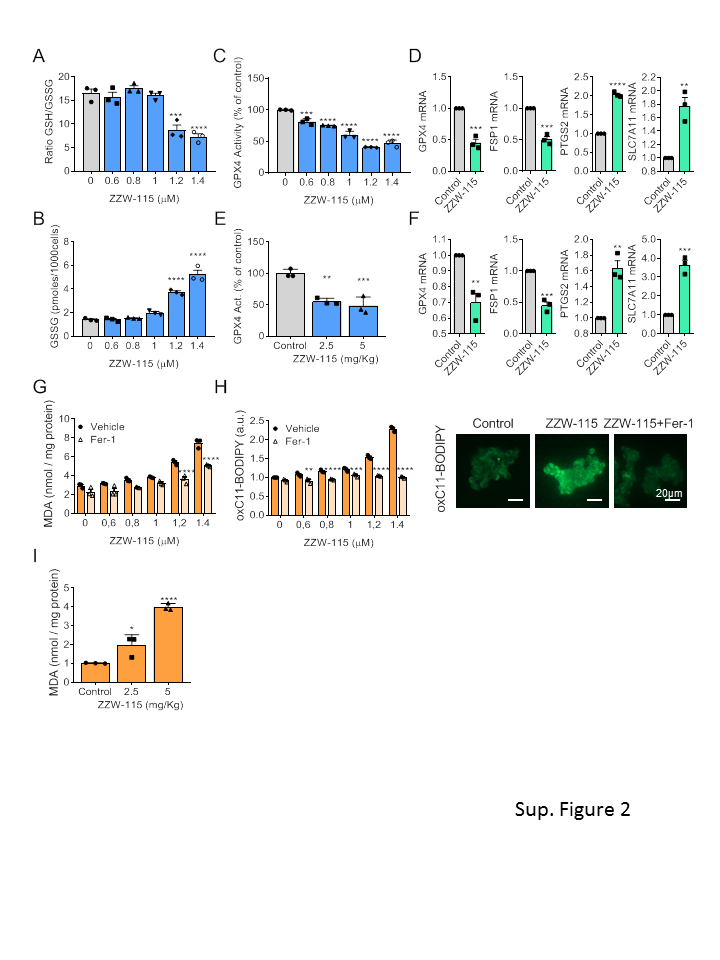

Supplement: Supplementary file 3 — Supp Figure 2 [file 41420_2021_662_MOESM3_ESM.tif]
